# Synthetic CT generation based on CBCT using improved vision transformer CycleGAN

**DOI:** 10.1038/s41598-024-61492-7

**Published:** 2024-05-20

**Authors:** Yuxin Hu, Han Zhou, Ning Cao, Can Li, Can Hu

**Affiliations:** 1 School of Computer and Software, Hohai University, Nanjing, 211100 China; 2 School of Electronic Science and Engineering, Nanjing University, NanJing, 210046 China; 3 Engineering Research Center of TCM Intelligence Health Service, School of Artificial Intelligence and Information Technology, Nanjing University of Chinese Medicine, Nanjing, 210023 China; 4 Department of Radiation Oncology, The Fourth Affiliated Hospital of Nanjing Medical University, Nanjing, 210013 China

**Keywords:** Medical imaging, Radiotherapy

## Abstract

Cone-beam computed tomography (CBCT) is a crucial component of adaptive radiation therapy; however, it frequently encounters challenges such as artifacts and noise, significantly constraining its clinical utility. While CycleGAN is a widely employed method for CT image synthesis, it has notable limitations regarding the inadequate capture of global features. To tackle these challenges, we introduce a refined unsupervised learning model called improved vision transformer CycleGAN (IViT-CycleGAN). Firstly, we integrate a U-net framework that builds upon ViT. Next, we augment the feed-forward neural network by incorporating deep convolutional networks. Lastly, we enhance the stability of the model training process by introducing gradient penalty and integrating an additional loss term into the generator loss. The experiment demonstrates from multiple perspectives that our model-generated synthesizing CT(sCT) has significant advantages compared to other unsupervised learning models, thereby validating the clinical applicability and robustness of our model. In future clinical practice, our model has the potential to assist clinical practitioners in formulating precise radiotherapy plans.

## Introduction

Image-guided radiotherapy(IGRT) technology serves as the cornerstone of accurate radiation therapy, facilitating the real-time monitoring of imaging changes in tumors and normal organs before and during treatment^1,2^. CBCT, as the primary imaging modality in IGRT, offers advantages such as lightweight and open architecture, along with reduced radiation exposure^3^. Consequently, CBCT finds extensive application in image-guided patient positioning and image registration within the clinical oncology radiation therapy workflows^4,5^. However, CBCT often suffers from noise and artifacts, making the process of segmentation more challenging^6,7^. Furthermore, CBCT images are less reliable compared to computed tomography (CT) when it comes to providing accurate CT/ED information^8^. These limitations hinder the clinical application of CBCT in adaptive radiotherapy. To overcome these shortcomings, we aim to enhance the quality of CBCT images and improve the accuracy of tumor treatment while ensuring better protection of organs at risk. This will be accomplished by generating synthetic CT (sCT) using the density from CT and the detailed anatomical information obtained from CBCT.

CT synthesis methods can be divided primarily into supervised and unsupervised approaches. Supervised learning relies on paired data and utilizes reconstruction loss to guide the network in generating sCT. The most representative techniques include fully convolutional networks (FCNs)^9,10^ and variations of the U-Net^11–15^. Dong et al.^10^ employed FCNs for the end-to-end nonlinear mapping from MRI to CT. Kida et al.^14^ applied a 2D U-Net deep convolutional neural network to the pelvis to synthesize CT from CBCT. Similarly, Li et al.^15^ utilized an enhanced U-Net structure with residual blocks to generate CT from CBCT in the head and neck region. Xie et al.^16^ presented a groundbreaking scatter artifact mitigation technique employing convolutional neural networks, complemented with a context-aware loss function, specifically designed to address artifact reduction in CBCT images. Liu et al.^17^ employed a U-Net architecture, trained on registered images, to generate intermediate CT representations from CBCT scans, with the objective of tackling artifact challenges and paving the way for subsequent unsupervised processing. Kihwan et al.^18^ introduced a self-supervised model, leveraging a blind input network to map partially obscured projections back to their original form, ultimately aiming to enhance the quality of CBCT denoised reconstructions. However, obtaining fully paired data in the clinical setting is challenging due to factors such as setup errors and organ movement. Consequently, supervised methods necessitate pre-processing to establish pairing prior to network training, as well as a high demand for precise image registration algorithms.

While unsupervised CycleGAN and its derivatives continue to hold relevance, alternative methods contribute significantly to image synthesis. Contrastive learning^19^ has emerged as a powerful technique, harnessing existing data to supervise model training in image generation tasks. Wang et al.^20^ developed a contrast learning-based approach for CBCT scatter correction, which employs a low-pass filter to remove unnecessary image details, isolates scatter projections, and then subtracts scattering artifacts from the original projections to produce improved images, with the aim of enhancing CBCT image quality. Their DCLGAN^21^ model further refines this concept by filtering strip artifacts, using inverse coordinate transformations to address ring artifacts, and generating corrected images by subtracting these artifacts from the original CT scans, striking a balance between artifact removal and detail preservation. Liu et al.^22^ introduced a weakly-supervised strategy that models the degradation between low-dose and normal-dose images in the latent space, focusing on generating high-quality low-dose images. Diffusion models, too, have garnered attention, with Li et al.^23^ frequency-guided diffusion model (FGDM) being a notable example. This model employs frequency-domain filters to guide the diffusion process, enabling more accurate cross-modal image transformations. Liu et al.^24^ presented a cascaded unconditional diffusion model that integrates low-dose CT images into the diffusion process, iteratively solving multiple maximum a posteriori problems to tackle image denoising effectively. Muzaffe et al.^25^ introduced SynDiff, an adversarial diffusion model that facilitates a gradual mapping of noise and source images to the target during conditional diffusion, demonstrating superior performance in MRI-CT conversion tasks. Lastly, Peng et al.^26^ employed a conditional denoising diffusion probabilistic model (DDPM) with a U-net architecture incorporating residual and attention blocks. This model converts Gaussian white noise samples into target CT images conditioned on CBCT, successfully achieving synthetic CT synthesis. This paper focuses on the classical unsupervised CycleGAN-based model.

Unsupervised learning differs from supervised learning in that it does not rely on reconstruction loss but instead leverages constraints rooted in its own network structure or other structures^27^. In the context of CT synthesis, the most common unsupervised models are cycle-consistent generative adversarial networks(CycleGAN)^28^ and its variations. Liu et al.^29^ employed a 3D CycleGAN with attention gates to generate CT images from non-paired pelvis data in CBCT synthesis. Harms et al.^4^ introduced residual blocks to the generator to achieve end-to-end mapping from CBCT to CT. Likewise, dense blocks^30–33^ are frequently integrated into the U-net architecture to efficiently extract and generate more accurate images. Moreover, meticulously designed loss functions are widely used to guide the process of image generation. Zeng et al.^34^ proposed a hybrid GAN that incorporates a weakly supervised mechanism for synthesizing CT from MRI when paired data is limited.

CycleGAN and its variant methods have been extensively explored for CT synthesis. However, there are still several challenges that need to be addressed. Previous studies have shown that the generator of CycleGAN and its variants are limited to preserving and transmitting local feature information, leading to low image quality and authenticity.

To address these issues, we propose a new unsupervised method called improved vision transformer CycleGAN (IViT-CycleGAN). (1) In the generator, we introduce a U-net framework based on vision transformer (ViT) to extract and preserve essential features and detailed information. The use of skip connections helps mitigate information loss. (2) For ViT, we further incorporate a deep convolutional network in the feed-forward neural network and combine it with the self-attention mechanism of the transformer. This integration aims to automatically attend to information from different positions in the image during generation, better understanding the global structure of the image, and focusing on regions with more details, resulting in clearer and more realistic generated images. (3) To improve the training stability of the model, we introduce gradient penalization, ensuring that the network weights do not undergo significant changes when there are minor variations in the model inputs. (4) To enhance the consistency between the generated images and the source images, we add an additional loss term in the generator loss. This loss captures the differences between the source and generated images.

## Methods

### CycleGAN models

**Figure 1:**
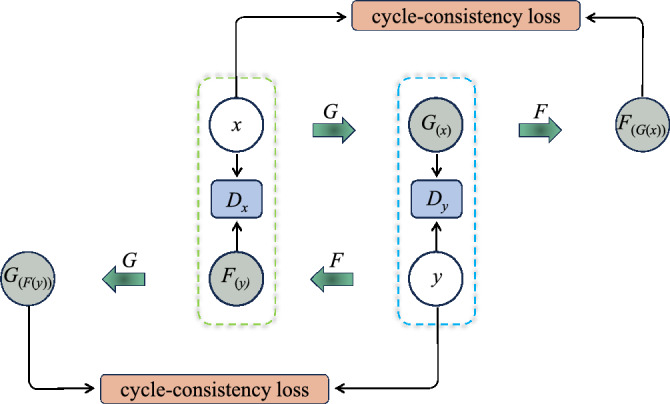
Schematic of the original CycleGAN model.

CycleGAN is shown in Fig. 1, where generator *G* aims to synthesize the input image *x* into a synthesized image $G_{(x)}$ that is highly similar in quality to the image of target domain *y*. The discriminator $D_{y}$ is designed to distinguish the real image in the target domain *y* from the image $G_{(x)}$ synthesized by the generator *G*^35^.

The discriminator performs loss updating by backpropagation:

1 $$\begin{aligned}&{\mathscr {L}}_{\textrm{disc},A}= {\mathbb {E}}_{x\sim B}\ell _{\textrm{GAN}}\left( {\mathscr {D}}_{A}\left( {\mathscr {G}}_{B\rightarrow A}(x)\right) ,0\right) +{\mathbb {E}}_{x\sim A}\ell _{\textrm{GAN}}\left( {\mathscr {D}}_A(x),1\right) , \end{aligned}$$

2 $$\begin{aligned}&\quad {\mathscr {L}}_{\textrm{disc},B}={\mathbb {E}}_{x\sim A}\ell _{\textrm{GAN}}\left( {\mathscr {D}}_B\left( {\mathscr {G}}_{A\rightarrow B}(x)\right) ,0\right) +{\mathbb {E}}_{x\sim B}\ell _{\textrm{GAN}}\left( {\mathscr {D}}_B(x),1\right) . \end{aligned}$$

where $\ell _{\textrm{GAN}}$ is the classification loss function. 0 and 1 are the class labels of the generated and real images respectively^36^.

The generators are updated by backpropagating loss from three sources: GAN loss, cycle-consistency loss, and identity-consistency loss^36^. Using ${\mathscr {G}}_{A\rightarrow B}$ as an example:

3 $$\begin{aligned}&{\mathscr {L}}_{\textrm{GAN},A}={\mathbb {E}}_{x\sim A}\ell _{\textrm{GAN}}\left( {\mathscr {D}}_{A}\left( {\mathscr {G}}_{A\rightarrow B}(x)\right) ,1\right) , \end{aligned}$$

4 $$\begin{aligned}&\quad {\mathscr {L}}_{\textrm{cyc},A}={\mathbb {E}}_{x\sim A}\ell _{\textrm{reg}}\left( {\mathscr {G}}_{B\rightarrow A}\left( {\mathscr {G}}_{A\rightarrow B}(x)\right) ,x\right) , \end{aligned}$$

5 $$\begin{aligned}&\quad {\mathscr {L}}_{\textrm{idt},A}={\mathbb {E}}_{x\sim A}\ell _{\textrm{reg}}\left( {\mathscr {G}}_{B\rightarrow A}\left( x\right) ,x\right) . \end{aligned}$$

And,

6 $$\begin{aligned}&{\mathscr {L}}_{\textrm{gen},A\rightarrow B}={\mathscr {L}}_{\textrm{GAN},A}+\lambda _{\textrm{cyc}}{\mathscr {L}}_{\textrm{cyc},A}+\lambda _{\textrm{idt}}{\mathscr {L}}_{\textrm{idt},A}, \end{aligned}$$

7 $$\begin{aligned}&\quad {\mathscr {L}}_{\textrm{gen},B\rightarrow A}={\mathscr {L}}_{\textrm{GAN},B}+\lambda _{\textrm{cyc}}{\mathscr {L}}_{\textrm{cyc},B}+\lambda _{\textrm{idt}}{\mathscr {L}}_{\textrm{idt},B}. \end{aligned}$$

Here, $\ell _{\textrm{reg}}$ can be any regression loss function, to harmonize the comparison experiments, we refer to CycleGAN^37^ parameter settings: $\lambda _{\textrm{cyc}}$=10 and $\lambda _{\textrm{idt}}$=0.5^36^.

### IViT-CycleGAN architecture

**Figure 2:**
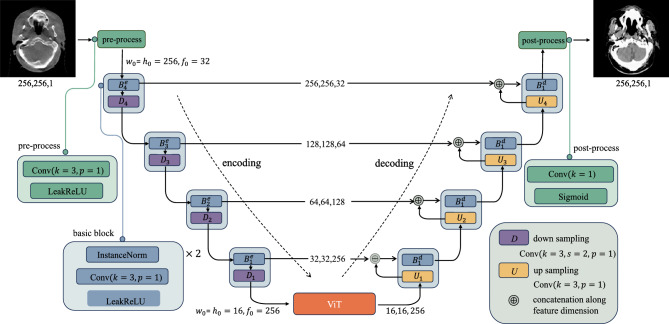
IViT-CycleGAN model generator section.

The original generator of CycleGAN can only retain and convey local feature information, lacking the ability to capture global features, thereby resulting in subpar image quality and authenticity. To address this limitation, this research incorporates a ViT-based U-net framework into the generator, as depicted in Fig. 2.

Firstly, the U-net architecture is employed to extract and retain crucial organizational features and detailed information, effectively resolving the issue of information loss through the utilization of skip connections. Subsequently, the self-attention mechanism of the transformer is employed to automatically prioritize information from various positions within the image during image generation, enhancing the comprehension of the global structure within organizational images. Lastly, a deep convolutional network is introduced into the feedforward neural network to concentrate on regions with more intricate details, resulting in clearer and more realistic generated images. Specifically, the coding path of U-net extracts features from the input through four layers of convolution and downsampling, and passes the extracted features from each layer to the corresponding layer of the decoding path through skip connections. In the encoding path of U-net, the preprocessing layer converts the image into a tensor with dimensions ($w_0$,$h_0$,$f_0$), and the preprocessed tensor halves the width $w_0$ and the height $h_0$ in each downsampled block while the feature dimension $f_0$ is doubled^36^.

**Figure 3:**
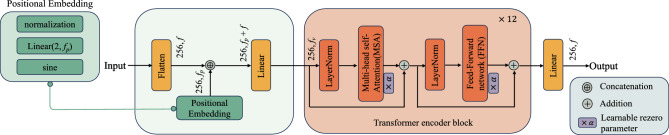
ViT module in IViT-CycleGAN.

For the ViT module, as shown in Fig. 3, ViT is composed primarily of a stack of transformer encoder blocks. To construct an input to the stack, the ViT first flattens an encoded To construct an input to the stack, the ViT first flattens an encoded image along the spatial dimensions to form a sequence of tokens. The token sequence has length ${w \times h}$, and each token in the sequence is a vector of length *f*. It then concatenates each token with its two-dimensional Fourier positional embedding of dimension $f_p$ and linearly maps the result to have dimension $f_v$^36^.

**Figure 4:**
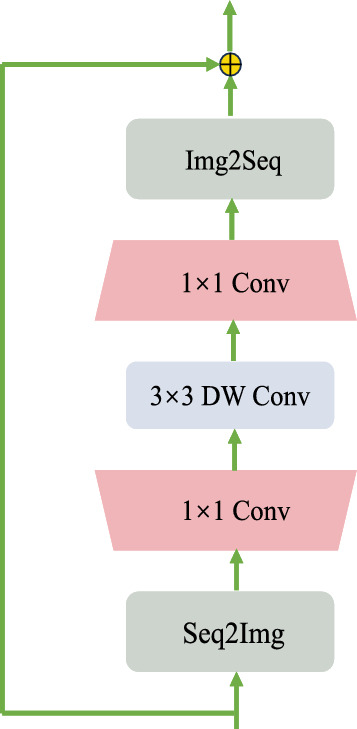
Depth-Wise Convolution Network. DW denotes depth-wise convolution. To cope with the convolution operation, the conversion between sequence and image feature map is added by Seq2Img and Img2Seq^38^.

For the feedforward neural network, as shown in Fig. 4, we use a deep convolutional network instead of the original fully connected layer.The input, i.e. a sequence of tokens is first reshaped to a feature map rearranged on a 2D lattice. Then two 1 $\times$ 1 convolutions along with a depth-wise convolution are applied to the feature map^38^. After that, the feature map is reshaped to a sequence of tokens which are used as by the self-attention of the network transformer layer.To improve the Transformer convergence, we adopt the rezero regularization scheme and introduce a trainable scaling parameter $\alpha$ that modulates the magnitudes of the nontrivial branches of the residual blocks. The output from the transformer stack is linearly projected back to have dimension *f* and unflattened to have width *w* and *h*. In this study, we use 12 transform encoder blocks^36^.

### Discriminator loss with gradient Penalty (GP)

To improve the training stability, we introduce a generalized GP^36,39^ form with the following ${\mathscr {D}}_{A}$ loss formula:

8 $$\begin{aligned}&{\mathscr {L}}_{\textrm{disc},A}^{\textrm{GP}}={\mathscr {L}}_{\textrm{disc},A}+\lambda _{\textrm{GP}}{\mathbb {E}}\left[ \frac{\left( \Vert \nabla _{x}{\mathscr {D}}_{A}(x)\Vert _{2}-\gamma \right) ^{2}}{\gamma ^{2}}\right] \end{aligned}$$

where ${\mathscr {L}}_{\textrm{disc},A}$ is defined as in Eq. 1, and ${\mathscr {L}}_{\textrm{disc},B}$ follows the same form. In our experiments, this $\gamma$-centered GP regularization provides more stable training and less sensitive to the hyperparameter choices^36^.

### Pixel-wise consistency loss

To improve the consistency of the generated and source images, we experiment with the addition of an extra term ${\mathscr {L}}_{\textrm{consist}}$^40^ to generator loss. This term captures the ${\mathscr {L}}_{\textrm{1}}$ difference between the downsized versions of the source and translated images. For example, for images of domain *A*:

9 $$\begin{aligned}&{\mathscr {L}}_{\textrm{consist},A}={\mathbb {E}}_A\ell _1\left( F({\mathscr {G}}_{A\rightarrow B}(a)),F(a)\right) \ \end{aligned}$$

where *F* is a resizing operator down to 32$\times$32 pixels (low- pass filter). We add this term to the generator loss with a magnitude $\lambda _{\textrm{consist}}$ for both domains^40^.

### Ethical statement

We confirmed that all methods were carried out in accordance with relevant guidelines and regulations, and informed consent for patients was waived by the Research Ethics Committee of the Nanjing Medical University. All experimental protocols and data in this study were approved by the Research Ethics Committee of the Nanjing Medical University. Approval number: NMUE2021301.

## Experiments

### Data acquisition

In this study, we test our proposed method in two datasets provided by a cooperative tertiary hospital.

#### H &N dataset

The CBCT and CT images were selected from 30 patients who were received volume modulated arc therapy(VMAT) in the head and neck (H &N) for nasopharyngeal and hypopharyngeal(NPC,HC) cancer from October 1,2021 and September 1,2023. The CT volumes were obtained with the dimensions 512$\times$512 on the axial plane with a pixel size of 0.625x0.625 $\mathrm{mm^{2}}$ and a slice thickness of 2.75 mm using GE discovery positioning system. The CBCT volumes were obtained using Elekta XVI Systems. The appiled images protocol were the following parameters: 200 degrees gantry rotation,$\mathrm 100kV_{p}$,10mA,10ms, and F0S20collimator. And images had a size of 384$\times$384 on the axial plane. Every patient contain with 1 planning CT volume taken in the positioning before treatment and 3 CBCT volumes every week taken between treatment. We randomly divided the training set and test set according to 8:2.

#### Chest dataset

The Chest dataset had the same acquisition time as the H &N dataset. The CT and CBCT parameters had few differences. It consisted of 30 patients, each with 1 planning CT volume taken before treatments and 3–5 CBCT volumes per week taken between treatments. The CT volumes were obtained with the dimensions $512\times 512$ on the axial plane with a pixel size of $0.625 \times 0.625$ mm^2^ and a slice thickness of 5 mm using GE discovery positioning system. And the CBCT images were acquired with the following parameter: 360 degrees gantry rotation,100 kVp, 10 mA, 10 ms, and F0M10 collimator. and the CBCT volumes were reconstructed at medium-resolution ($1\times 1 \times 1\,\mathrm{mm^{3}}$ voxels) on a $410 \times 410 \times 120$ matrix. We randomly divided the training set and test set according to 8:2.

### Data processing

During the scanning process of CBCT and CT, non-human structures (such as treatment beds, fixation devices, and masks) are captured in the resulting images. These structures not only impede model training speed but also compromise the quality of synthesized images. To mitigate these issues, denoising is essential to eliminate the interference of irrelevant information prior to model training. In this study, the outlines of CT contours manually annotated by doctors serve as masks. These masks are subsequently multiplied with the corresponding CT images to generate clean CT images. Likewise, the masks are also multiplied with the CBCT images to produce clean CBCT images suitable for training purposes. Distinctive characteristics distinguish CBCT from CT, encompassing variations in imaging hardware, clinical protocols, and scanning configurations. Matching scans from the same patient often presents a challenge due to the inherent differences. Recognizing the stability of organ positions and reduced tissue mobility during data acquisition, we leveraged the open-source advanced normalization tools (ANTs) for affine registration, with the primary objective of ensuring alignment between each CBCT and CT pair for the purpose of model test.

### Evaluation

To accurately compare the similarity between the sCT images generated by different models and the CT images, we introduced quantitative evaluation metrics such as mean absolute error (MAE), peak signal-to-noise ratio (PSNR), and structural similarity index (SSIM). A lower MAE value indicates less difference between sCT and CT, resulting in more realistic image generation. Conversely, higher values of PSNR and SSIM indicate greater similarity between sCT and CT, leading to higher construction quality and more realistic images. These metrics are defined as:

10 $$\begin{aligned} MAE=\frac{1}{n}\sum _{i=1}^{n}|x_{i}-G_{CBCT\rightarrow CT}(y_{i})|\ \end{aligned}$$

*n* denotes the number of testing slices, and *x* and *y* denote the CT and the CBCT, respectively.The CBCT generates the predicted CT after the CBCT generator $G_{CBCT\rightarrow CT}$ , and then calculates the the absolute value error with the CT.

11 $$\begin{aligned} PSNR=20log_{10}\frac{MAX_{rCT}}{MSE}\ \end{aligned}$$

$MAX_{rCT}$ denotes the maximum pixel value in the sCT, and MSE is the mean square error. a larger PSNR indicates a higher similarity between the generated CT and the real CT, which means that the quality of the generated CT is better.

12 $$\begin{aligned} SSIM=\frac{(2\mu _{x}\mu _{y}+c_{1})(2\sigma _{xy}+c_{2})}{(\mu _{x}^{2}+\mu _{y}^{2}+c_{1})(\sigma _{x}^{2}+\sigma _{y}^{2}+c_{2})}\ \end{aligned}$$

where *x* and *y* represent the CT and the fake-CT generated by the CBCT after the generator $G_{CBCT\rightarrow CT}$, respectively, and $u_{x}$ and $u_{y}$ represent the mean of *x* and *y*, $\sigma _{x}^{2}$ and $\sigma _{y}^{2}$ represent the variance of *x* and *y*, and $\sigma _{xy}$ represents the variance of *x* and covariance of *y*, while $c_{1}$ and $c_{2}$ are the two constants used to maintain stability.The value of SSIM ranges from $-1$ to 1, and the larger the value, the more similar the two images are.

### Network training

**Figure 5:**
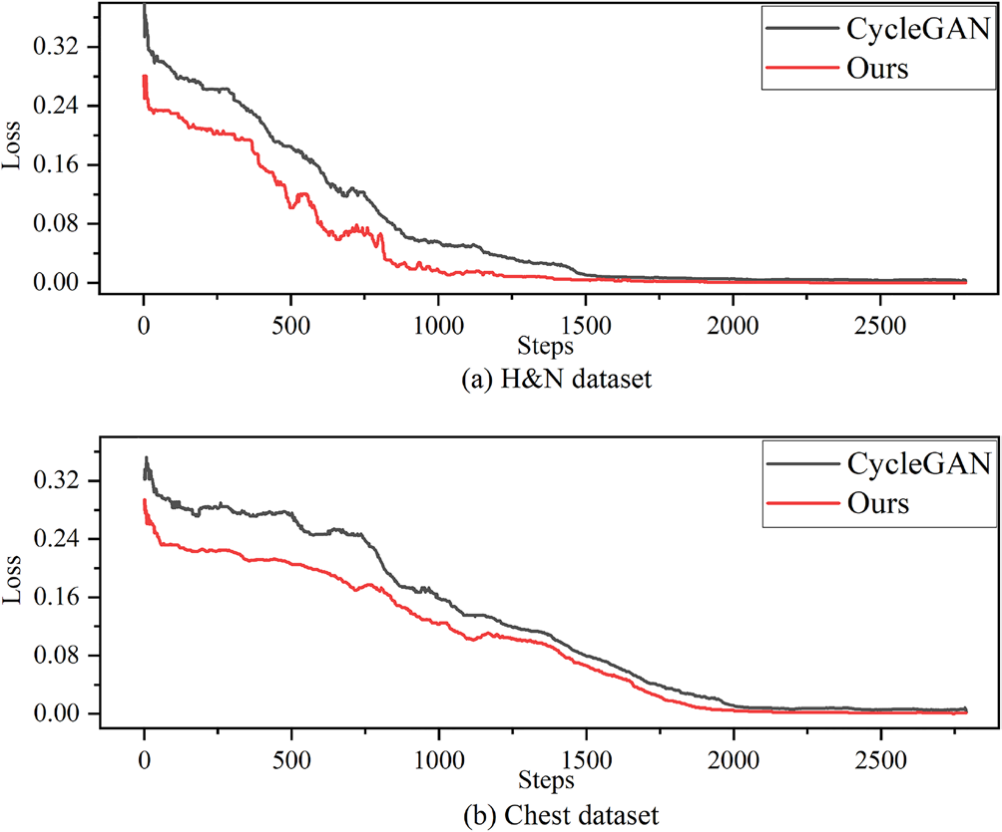
Plot of loss function vs. number of iterations on both datasets.

In the experiments, all images are normalized to [$-1$,1] and resized to 256 $\times$ 256. we train the generator five times and then train the discriminator once. Its parameters are set to *epoch* = 200 and *batch size*= 5. The other comparison methods are implemented based on the code and details provided by the authors and have the same hyperparameter settings as ours. All algorithms in this study were implemented on a Linux system equipped with four NVIDIA Tesla V100s using Python 3.6 (https://www.python.org/downloads/release/Python-362/) and Tensorflow 1.14 (https://tensorflow.google.co.uk/versions) implementations. Figure 5 shows a plot of the discriminator loss function versus the number of iterations on the two datasets, H &N and Chest, which shows that the proposed method in this paper (Ours) has faster convergence and is better trained.

## Result and discussion

### Comparison of different methods

Tables 1 and 2 present a comparison of quantitative results between our method and several contrast algorithms. Our proposed method demonstrates superior performance to CycleGAN and its variations in all three evaluation metrics mentioned above on the H &N dataset. This is attributed to the introduction of an improved ViT-based U-net framework. This framework enables the extraction and preservation of essential features and detailed information, automatic focus on information from different positions in the images, better comprehension of the global structure of the images, and emphasis on regions with more details. Consequently, the generated images are clearer and more realistic. Furthermore, we apply our method to the Chest dataset without altering any parameters and report the experimental results in Table 2. Our method exhibits considerable advantages on the Chest dataset as well, outperforming CycleGAN and its variations in the evaluated metrics. The key factor behind this success is the incorporation of GP and additional pixel-wise consistency loss, which enhance the stability and robustness of the model. These experiments validate the applicability of our method not only to the head and neck region but also to other parts of the body.

**Table 1:** Comparison of metrics of different methods on the H &N dataset.

**Method**	**MAE** $\downarrow$	**PSNR** $\uparrow$	**SSIM** $\uparrow$
CycleGAN^4^	15.13	29.1536	0.9712
DualGAN^41^	18.59	28.5969	0.9624
AttentionGAN^29^	25.68	27.1325	0.9513
RegGAN^42^	14.74	29.8562	0.9742
ADCycleGAN^35^	13.56	30.1598	0.9762
Ours	**10.94**	**31.8567**	**0.9834**

Significant values are in bold.

**Table 2:** Comparison of metrics of different methods on the Chest dataset.

**Method**	**MAE** $\downarrow$	**PSNR** $\uparrow$	**SSIM** $\uparrow$
CycleGAN^4^	20.89	27.6615	0.9612
DualGAN^41^	27.46	25.3512	0.9429
AttentionGAN^29^	23.29	26.1259	0.9526
RegGAN^42^	17.36	28.1523	0.9631
ADCycleGAN^35^	15.12	28.4563	0.9649
Ours	**13.46**	**29.1289**	**0.9715**

Significant values are in bold.

### Ablation studies for IViT-CycleGAN

To thoroughly assess the efficacy of each module, we employed CycleGAN-ViT as our backbone and conducted a module stacking analysis to gauge their individual impact on the overall performance. The abbreviations used are as follows: Depth-Wise Convolution Network (DCN), Discriminator Loss with Gradient Penalty (GP), and Pixel-wise Consistency Loss (PL). The results from the H &N dataset are compiled in Table 3, while those from the Chest dataset are presented in Table 4.

The experimental findings substantiate the contributions of each module to the overall performance. DCN due to its inherent local properties, complements the self-attention mechanism in ViT, enabling it to engage in both global and regional information exchange. This local focus facilitates the extraction of finer details, resulting in more vivid and realistic generated images. GP, through regularization controlled by the parameter $\beta$, improves model stability during training. PL, by measuring the L1 difference between source and generated images, enhances consistency, thereby enhancing image generation quality. A comparative analysis of metrics, including MAE, PSNR and SSIM, reveals that DCN exhibits the most significant performance boost compared to GP and PL.

**Table 3:** Quantitative results for ablations based on CycleGAN-ViT in H &N dataset.

**Method**	**MAE** $\downarrow$	**PSNR** $\uparrow$	**SSIM** $\uparrow$
CycleGAN-ViT	14.88	29.2561	0.9726
CycleGAN-ViT-DCN	13.12	29.8626	0.9768
CycleGAN-ViT-GP	13.48	29.3915	0.9752
CycleGAN-ViT-PL	14.64	29.3321	0.9744
CycleGAN-ViT-DCN-GP	11.78	31.4931	0.9825
CycleGAN-ViT-DCN-PL	12.62	30.3524	0.9788
CycleGAN-ViT-GP-PL	12.33	31.0162	0.9808
Ours	**10.94**	**31.8567**	**0.9834**

Significant values are in bold.

**Table 4:** Quantitative results for ablations based on CycleGAN-ViT in Chest dataset.

**Method**	**MAE** $\downarrow$	**PSNR** $\uparrow$	**SSIM** $\uparrow$
CycleGAN-ViT	16.88	27.9823	0.9633
CycleGAN-ViT-DCN	15.02	28.5621	0.9684
CycleGAN-ViT-GP	15.35	28.3589	0.9667
CycleGAN-ViT-PL	16.12	28.0124	0.9641
CycleGAN-ViT-DCN-GP	13.79	28.9652	0.9708
CycleGAN-ViT-DCN-PL	14.33	28.6814	0.9699
CycleGAN-ViT-GP-PL	14.01	28.7714	0.9701
Ours	**13.46**	**29.1289**	**0.9715**

Significant values are in bold.

### Visualization

**Figure 6:**
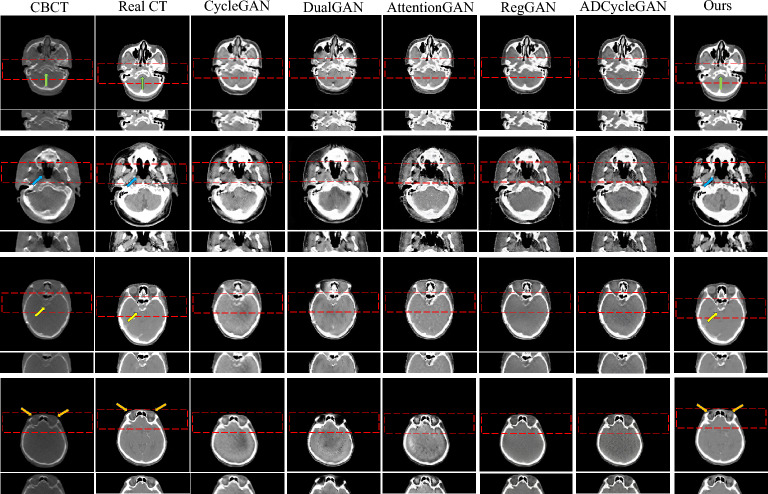
Visualization of sCT generation details on the H &N dataset test set.

In addition to quantifying the sCT using the aforementioned evaluation indicators, we incorporate visualization techniques to explore the results from various perspectives and validate the effectiveness of our proposed method by comparing the outputs of different models. In Fig. 6, we present a comparison of synthesis results obtained from six algorithms, namely CycleGAN, DualGAN, AttentionGAN, RegGAN, ADCycleGAN, and Ous , in H &N patients. These results showcase the generated images of the cervical Bone (marked by green arrows), Nasopharynx (marked by blue arrows), Pituitary (marked by yellow arrows), and Eyes (marked by orange arrows) in a sequential left-to-right and top-to-bottom manner. Upon closer examination of the magnified images, it is evident that the alternative algorithms yield images with increased noise levels and significant loss of lesion details. In contrast, our proposed method generates images characterized by minimal noise, enhanced details accuracy, and closer resemblance to Real CT results. This remarkable outcome can primarily be attributed to the incorporation of the ViT-based U-net framework, which excels in feature extraction while preserving crucial detailed information. The framework also demonstrates improved comprehension of the image’s global structure, resulting in the production of images that are significantly clearer and more realistic.

**Figure 7:**
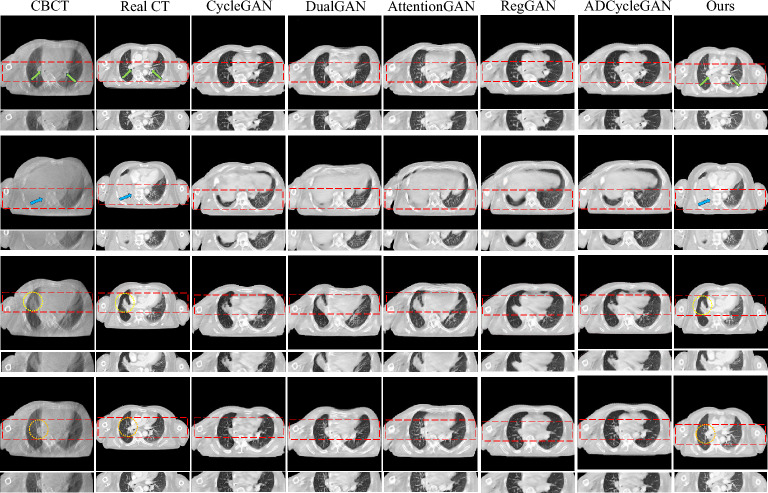
Visualization of sCT generation details on the Chest dataset test set.

**Figure 8:**
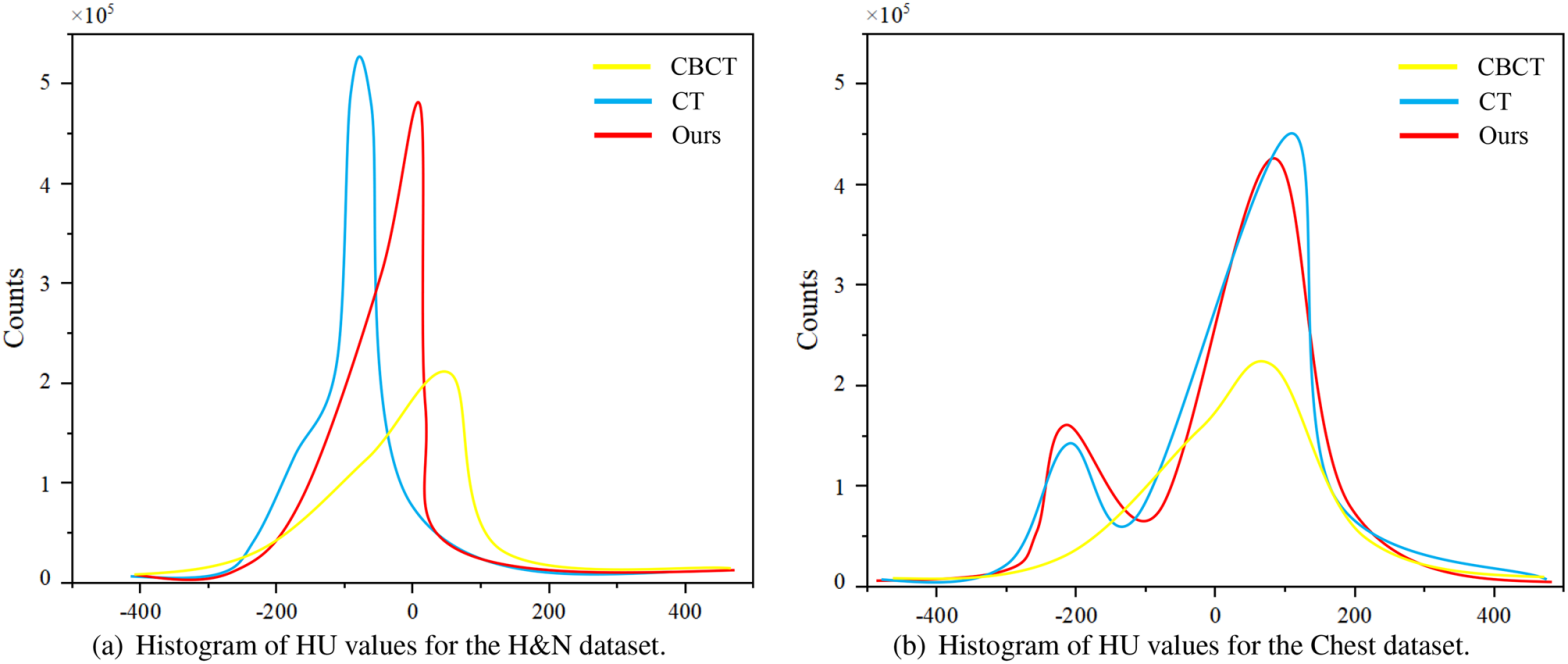
Histograms of HU values for the two types of datasets.

**Figure 9:**
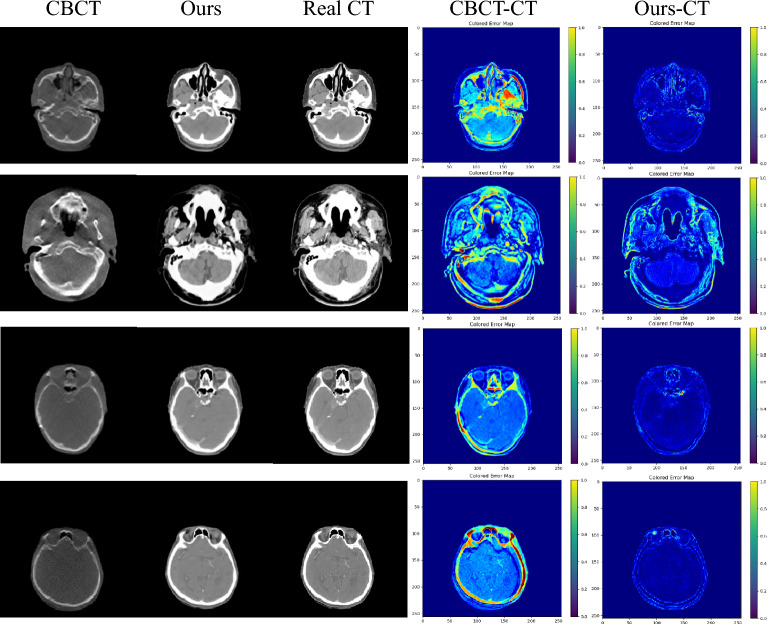
Error plots of CBCT vs. CT and the method of this paper vs. CT under the H &N dataset.

**Figure 10:**
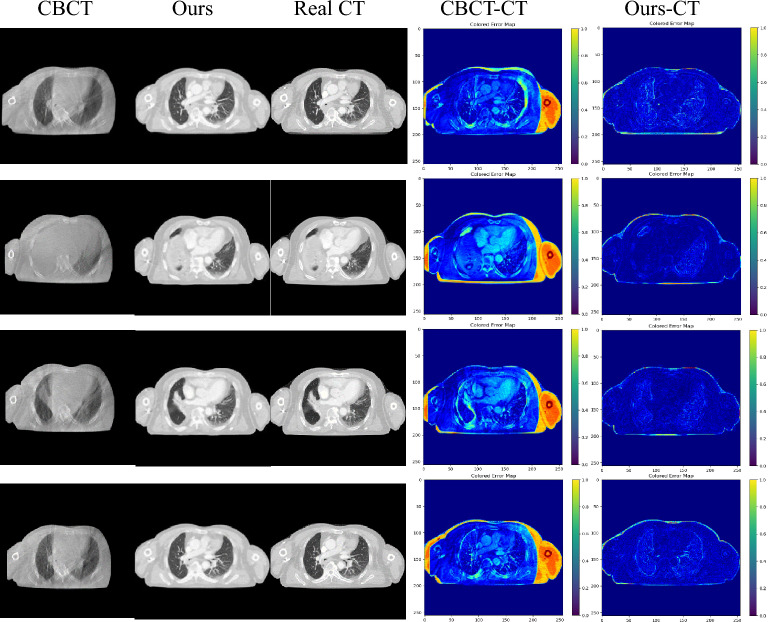
Error plots of CBCT vs. CT and the method of this paper vs. CT under the Chest dataset.

We also conducted a comparative analysis of the synthesis results obtained from the six algorithms on the Chest dataset, as shown in Fig. 7. The first and second rows illustrate the Bronchi (highlighted by green arrows) and the Sternum (highlighted by blue arrows) as the representative anatomical structures, respectively, while the third and fourth rows depict the lung tumor regions. A thorough examination of the partially enlarged images reveals that our method applied to the Chest dataset produces consistent outcomes with those observed in the H &N dataset. Specifically, the generated images exhibit diminished noise, enhanced detail accuracy, and a greater resemblance to Real CT scans. Notably, our proposed algorithm demonstrates a more striking similarity to Real CT scans in the tumor regions, which proves instrumental in discerning changes within the tumor areas and offering valuable image references for adaptive radiotherapy. The Hounsfield Units (HU) CT values, reflecting tissue density and X-ray absorption, were analyzed in the test set slices ranging from $-500$ to 500 HU. Figure 8 exhibits a comparative histogram of HU values for our method and CBCT, revealing that the curve shapes and peak positions of our approach more closely resemble those of real CT scans, suggesting a certain level of accuracy in the generated sCT. Figures 9 and 10 present difference plots comparing our method, CBCT, and ground truth CT. Utilizing a rainbow color mapping, with blue indicating minimal difference and red indicating maximum, the plots demonstrate that the discrepancies between our method and CT are significantly smaller than those between CBCT and CT, indicating that the sCT generated in this study achieves a CT-like quality to a considerable extent.

**Figure 11:**
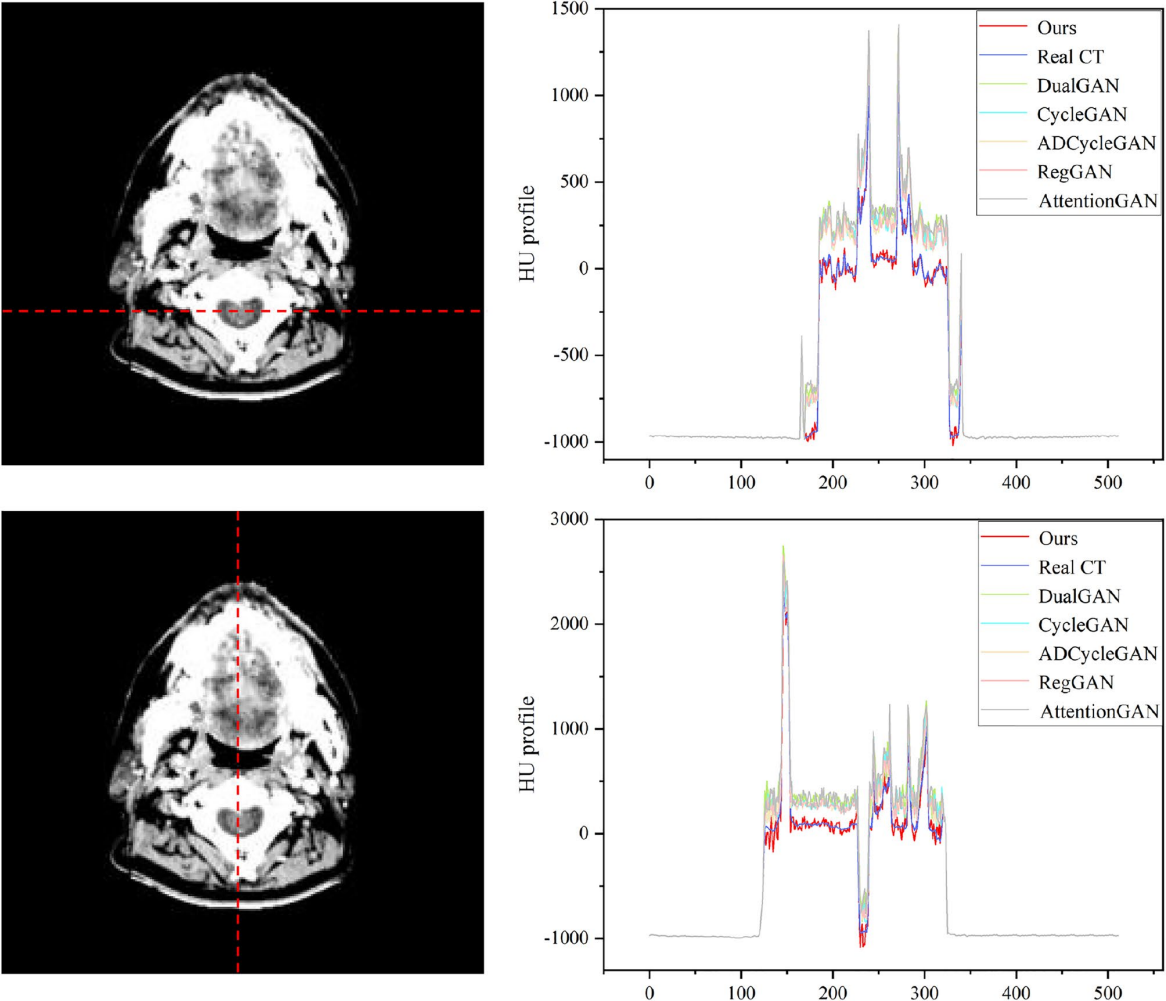
Visualization of CT value distribution on the H &N dataset test set.

**Figure 12:**
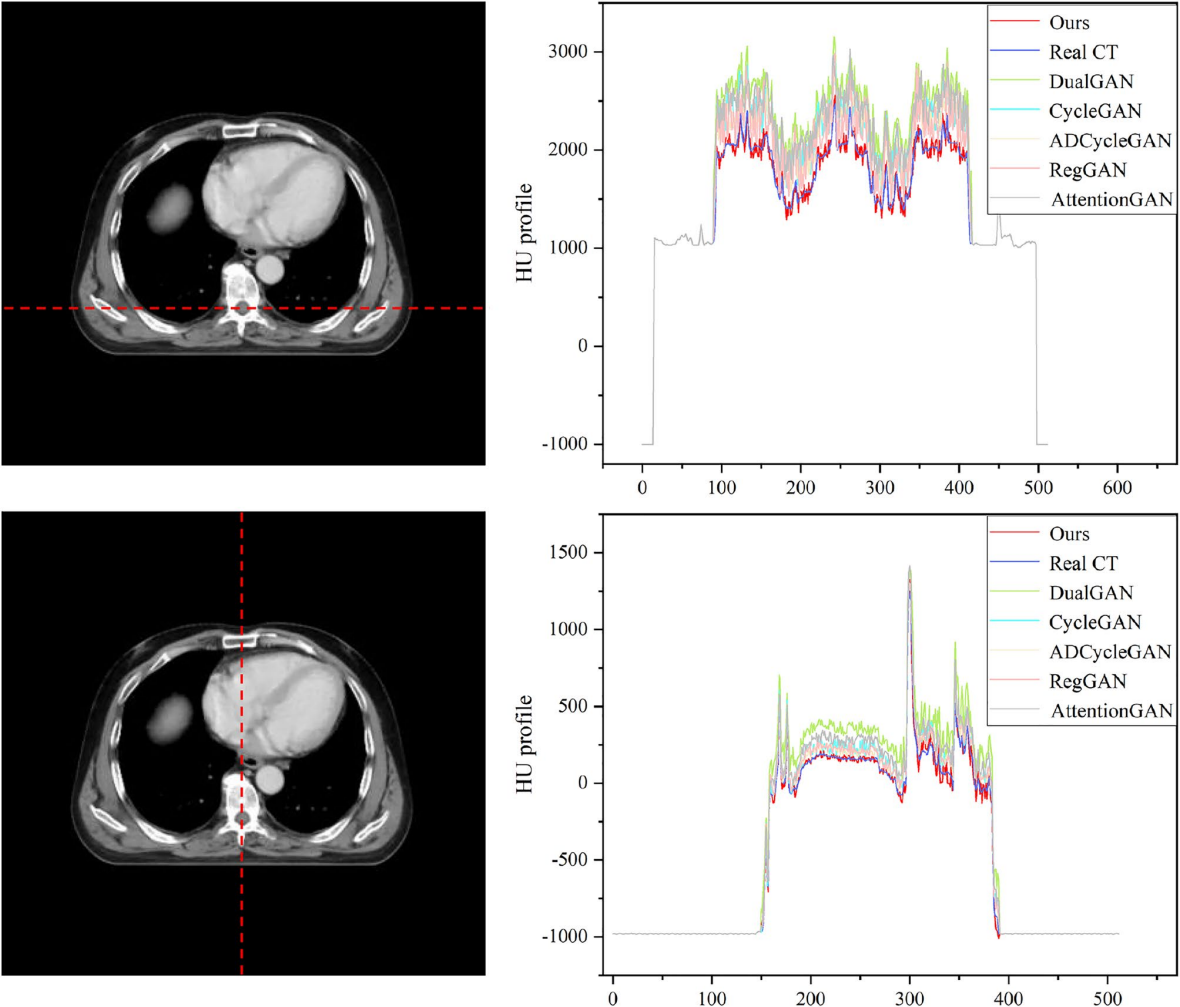
Visualization of CT value distribution on the Chest dataset test set.

In addition to comparing the generated details, we also assess the performance of different methods using CT values. We use each pixel on the vertical and horizontal axes as a unit and calculate the average CT value at each pixel of all the data in the entire test set. Figures 11 and 12 showcase the distribution of average CT values across the vertical and horizontal axes. The x-axis denotes the pixel position, while the y-axis represents the average pixel value. The purple curve corresponds to the CT value distribution curve of Real CT, the red curve represents our proposed method, and the remaining colors indicate other methods. The outcomes highlight the congruence of CT value distribution trends between Real CT and the other five methods. Notably, our CT value distribution curve bears a stronger resemblance to Real CT when juxtaposed with the curves of the other five methods. Meanwhile, the CT values obtained from the other methods slightly surpass those of Real CT. In essence, our method generates sCT images that closely approximate Real CT, thus rendering them more authentically realistic.

### Visualization of the synthetic CT 3D reconstruction

**Figure 13:**
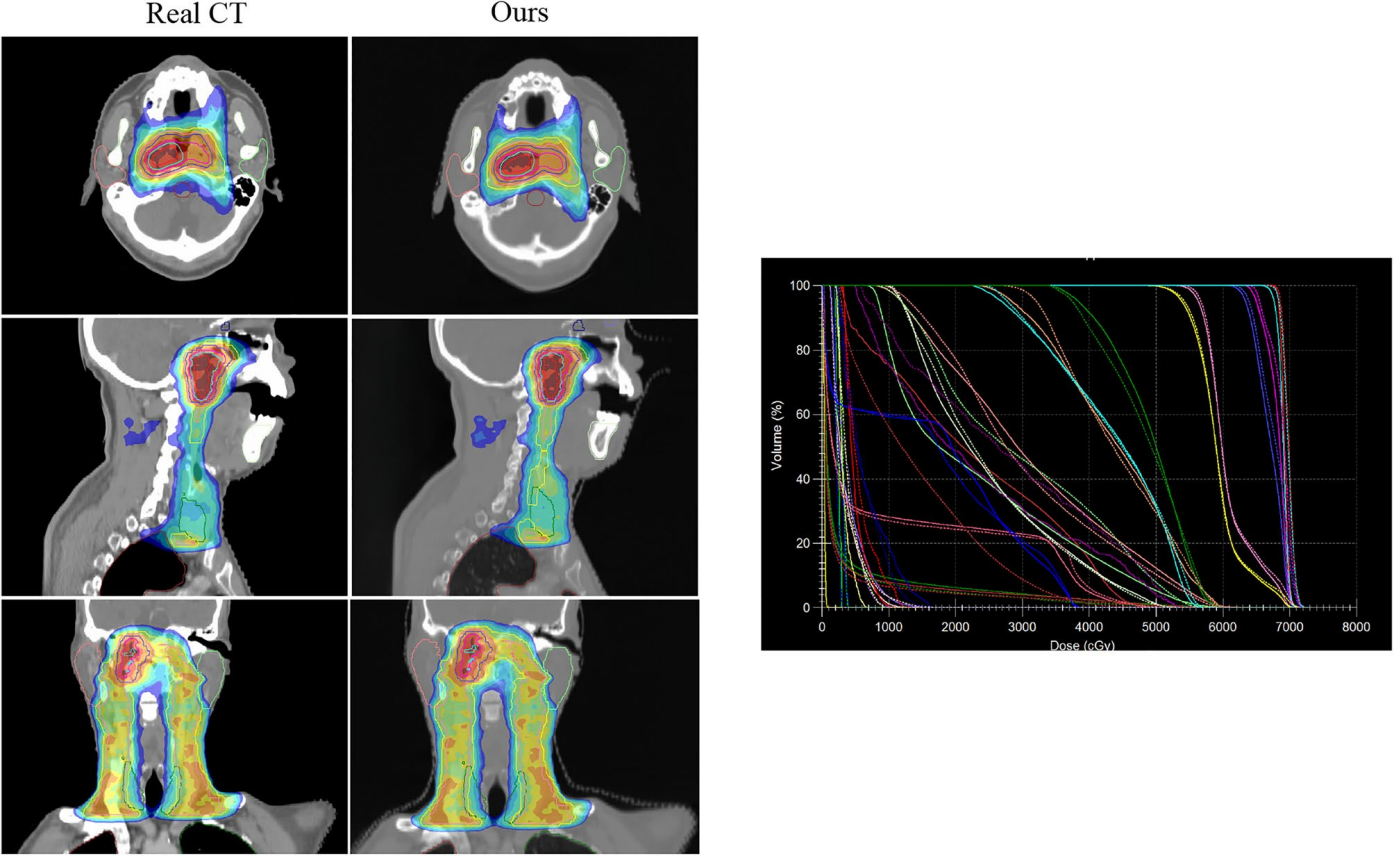
3D reconstruction visualization of the H &N dataset. From top to bottom, representing axial, sagittal and coronal planes(left). Patient DVH differences, where the solid line is Real CT and the dashed line is sCT (right).

**Figure 14:**
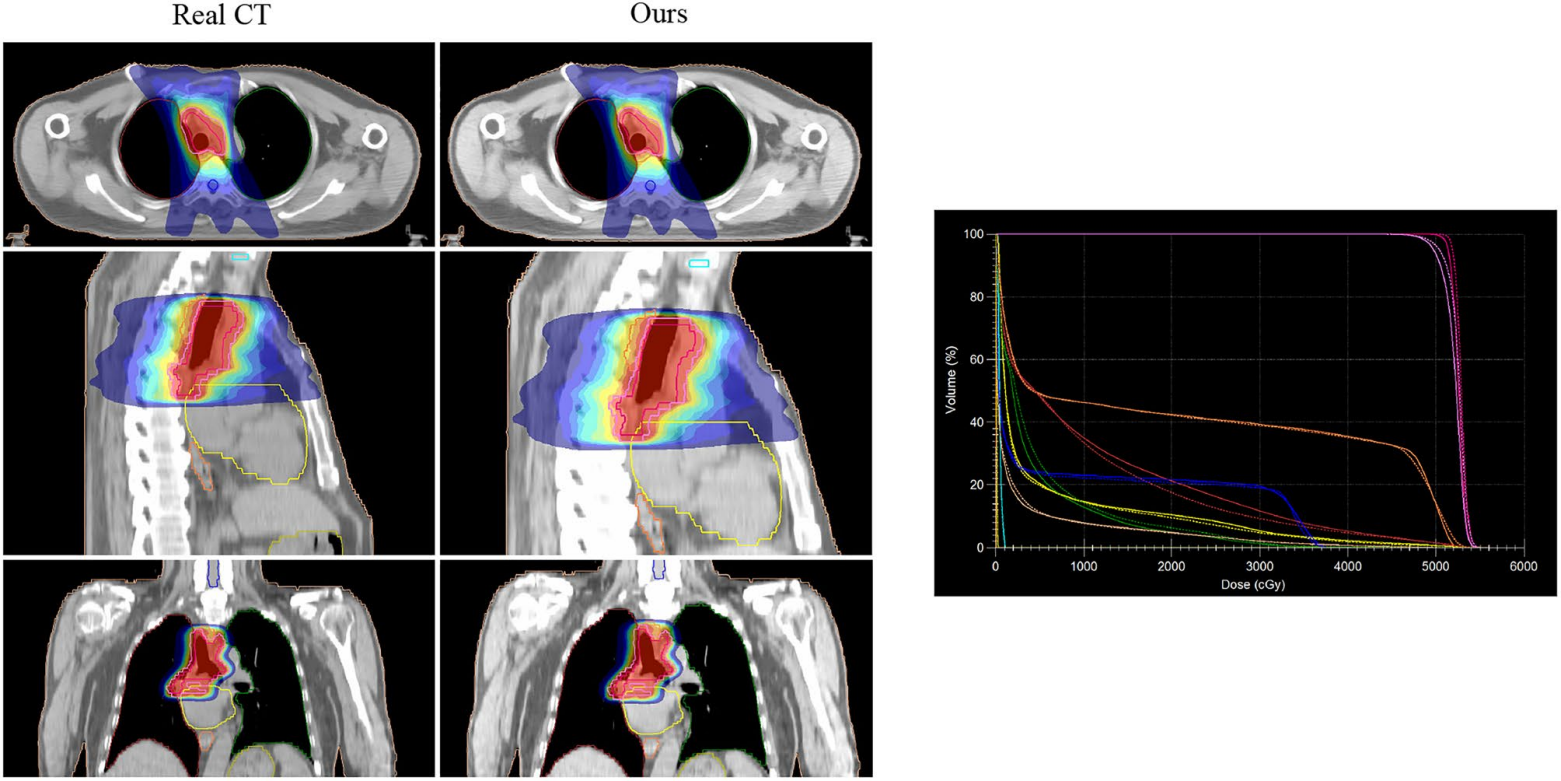
3D reconstruction visualization of the Chest dataset. From top to bottom, representing axial, sagittal and coronal planes(left). Patient DVH differences, where the solid line is Real CT and the dashed line is sCT (right).

The CT’s imaging process, characterized by its distinct mechanism, yields two-dimensional data in the form of X-ray-derived slices. In clinical settings, three-dimensional reconstructions are crucial for multi-faceted analysis of patient lesions, facilitating accurate diagnosis and treatment. To evaluate the fidelity of our sCT, we conducted 3D reconstructions, focusing on the consistency across dimensions. Figures 13 and 14 illustrate the results for the H &N and Chest datasets, featuring axial, sagittal, and coronal perspectives. Our method generates the axial view, while the sagittal and coronal views are reconstructed from the sCT. The reconstructed slices from these datasets evidence that the generated sCT successfully retains the original anatomical integrity, ensuring a consistent representation of internal organ structures. For the Dose and Volume Histogram (DVH) on the right side of Figs. 13 and 14, where the solid line represents Real CT and the dashed line represents the Ours method, it can be seen that the close proximity of our method to the real clinical dose distribution validates the clinical applicability of our method.

### Dose calculation

**Figure 15:**
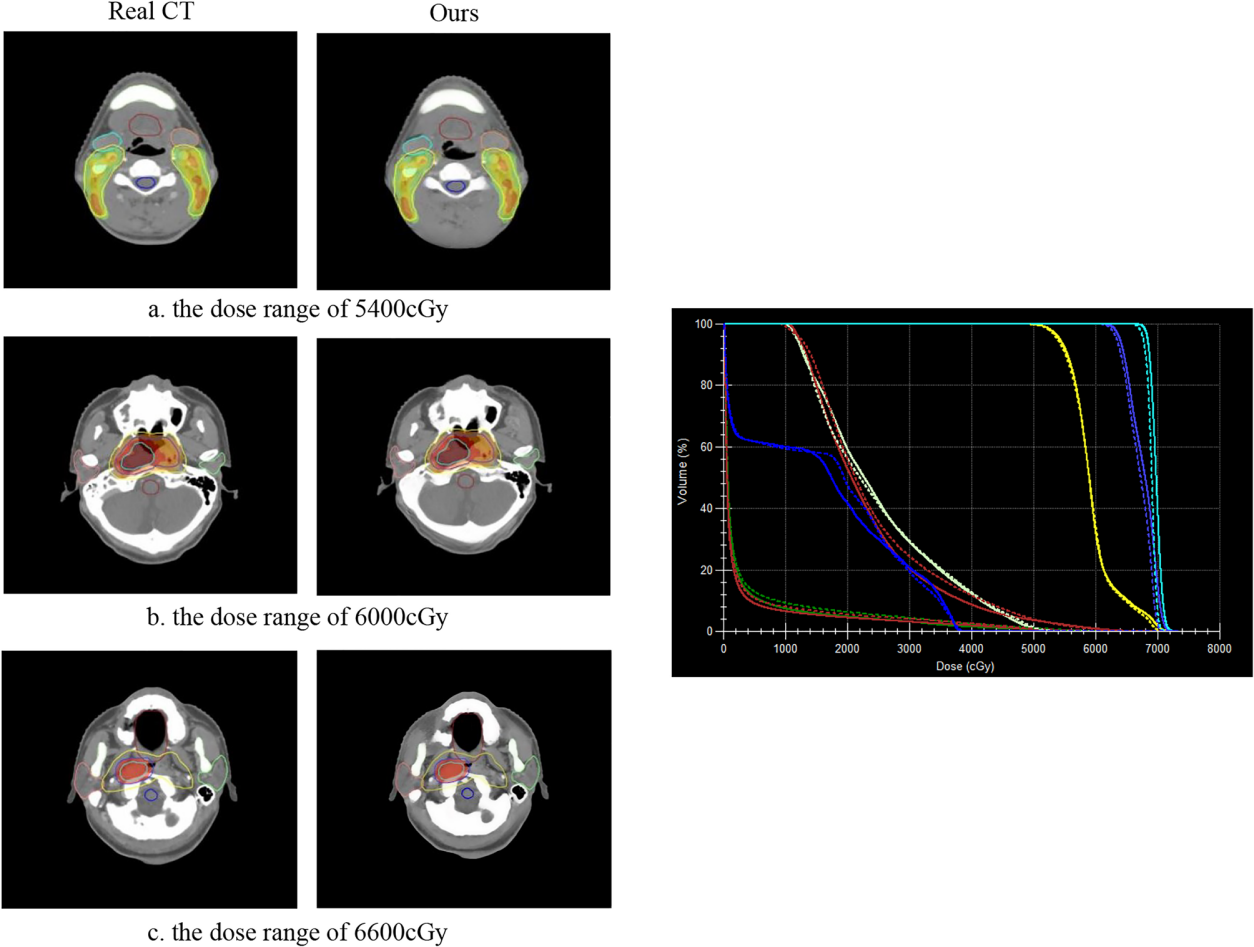
Differences between our method and Real CT for different dose distributions on the H &N dataset (left). Patient DVH differences, where the solid line is Real CT and the dashed line is sCT (right).

**Figure 16:**
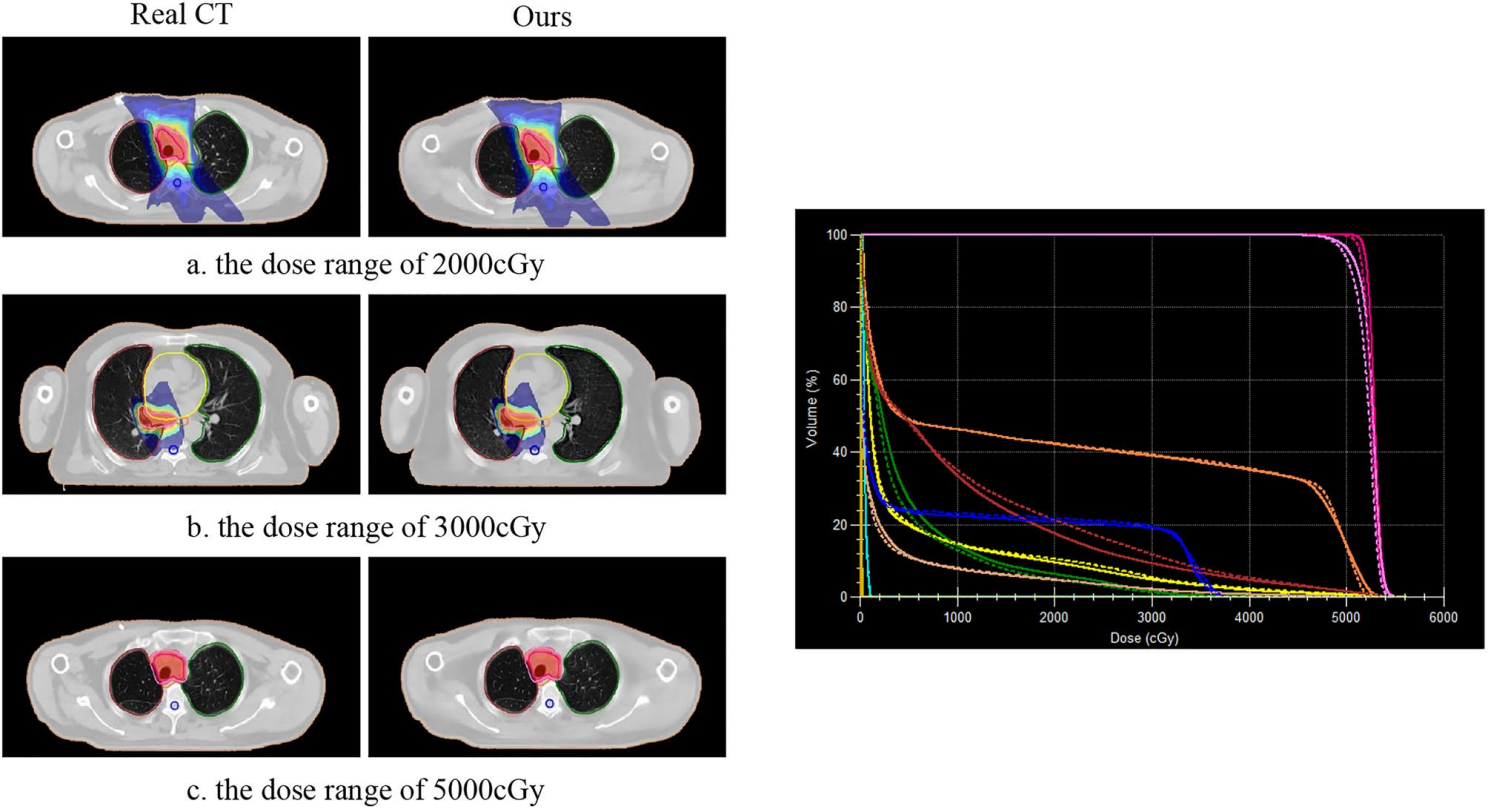
Differences between our method and Real CT for different dose distributions on the Chest dataset (left). Patient DVH differences, where the solid line is Real CT and the dashed line is sCT (right).

The primary purpose of sCT is to serve as a foundation for subsequent clinical tasks, particularly dose calculation. Hence, dose calculation offers the most precise approach to verifying the effectiveness of sCT generation and its clinical suitability. To this end, we conducted a comparison between the sCT generated by our method and the Real CT across different dose levels. The resulting discrepancies were then visualized in three-dimensional displays, as presented in Figs. 15 and 16. On the left side of Fig. 15, the differences between our method and the Real CT treatment plan for a nasopharyngeal cancer patient under different dose distributions are shown. On the right side of Fig. 15, the differences in DVH for the patient are displayed, with the solid line representing Real CT and the dashed line representing our method. It can be observed from the left side of Fig. 15 that our method closely approximates the actual clinical dose distribution under different dose distributions. Examination of the DVH on the right side of Fig. 15 reveals nearly no disparity in the preventive dose for the nasopharyngeal target area and lymphatic drainage area. The experiment successfully validates the clinical applicability of our method. Furthermore, for Chest patients, we observed the discrepancies between our method and Real CT in sCT under different dose distributions. Based on the 3D dose distribution and DVH in Fig. 16, it can be concluded that there is also a slight disparity in the dose received by the target area and lung tissue, thereby further confirming the clinical applicability and robustness of our method.

## Discussion

This research introduces IViT-CycleGAN, an unsupervised learning model designed to synthesize sCT from CBCT data. The selection of CycleGAN is driven by the practical challenge of obtaining paired CBCT and CT scans in clinical settings. Our approach enhances the original CycleGAN by incorporating a ViT-based U-Net generator, which effectively extracts and retains vital features and fine details. To further refine image generation, we integrate a deep convolutional network within the feedforward neural network, leveraging the Transformers’ self-attention mechanism to enable automatic focus on diverse image regions, thereby improving global understanding and enhancing detail localization. A gradient penalty is introduced to ensure more stable training, and an additional loss term is added to the generator’s objective function to capture discrepancies between the source and generated images.

Our model exhibits superior quantitative performance compared to prevailing unsupervised learning techniques, achieving state-of-the-art evaluation metrics across both datasets. Comprehensive ablation studies, detailed in Tables 3 and 4, consistently reveal the positive impact of our proposed modules on the model’s overall efficacy. Of particular note, the DCN module stands out with a more substantial boost, attributed to its inherent local characteristics that are effectively modeled by the self-attention mechanism in ViT. This integration enables ViT to engage in both global and local information exchange, thereby enhancing its capabilities.

In visual assessments, we rigorously tested our model’s superiority through extensive experiments. As depicted in Figs. 6 and 7, our model-generated images exhibit reduced noise and enhanced detail, closely resembling authentic CT scans. For the H &N dataset’s first row, our sCT exhibits the closest resemblance to real CT at the conus region (green arrow), with ADCycleGAN and RegGAN also displaying comparable performance. However, in the nasal cavity, ADCycleGAN and RegGAN differ significantly from the real CT in shape. CycleGAN, DualGAN, and AttentionGAN exhibit larger discrepancies, characterized by blurred details and excessive noise in the conus area. In the nasopharynx (blue arrow) of the second row, ADCycleGAN, RegGAN, CycleGAN, and DualGAN present similar shapes with minor differences from the real CT, such as blurred boundaries and missing details. Our sCT stands out with clear details, while AttentionGAN performs the least favorably. In the pituitary region (yellow arrow) of the third row, our sCT most closely matches the real CT, with distinct boundaries and minimal shape variations. ADCycleGAN, RegGAN, and CycleGAN lose some details, and eye distortion is prominent. DualGAN and AttentionGAN generally underperform. In the eye region (orange arrow) of the fourth row, ADCycleGAN, RegGAN, and CycleGAN exhibit minimal differences from the real CT, but brain tissue distortion is severe. Our sCT excels, whereas DualGAN and AttentionGAN lag behind. For the Chest dataset, in the bronchial bifurcation (green arrow), ADCycleGAN, RegGAN, and CycleGAN exhibit smaller differences from the real CT, but overall image detail is lacking. Our sCT stands out, while DualGAN and AttentionGAN falter. In the conus region (blue arrow) of the second row, ADCycleGAN and RegGAN show less shape deviation compared to CycleGAN, DualGAN, and AttentionGAN, which exhibit the greatest discrepancies. Our sCT is the closest to the real CT in this region. In the lung tumor area (yellow dashed circle), ADCycleGAN and RegGAN have similar shapes to the real CT, but they lack information in the heart and conus regions. Our sCT outperforms others, with CycleGAN, DualGAN, and AttentionGAN performing the worst. In the lung tumor area of the fourth row (orange dashed circle), ADCycleGAN and RegGAN have slightly less shape deviation from the real CT, but they severely lack heart information. Our sCT demonstrates superior clarity with minimal distortion, while CycleGAN, DualGAN, and AttentionGAN remain inferior. The HU value histograms in Fig. 8 reveal that our method’s curve shape and peak are closer to the real CT, indicating a certain level of fidelity. Figures 9 and 10 illustrate the difference plots between CBCT, our method, and CT for both datasets. The difference plots reveal that our method exhibits significantly smaller discrepancies compared to CBCT, indicating that the synthetic sCT generated by our approach approaches the CT standard to a certain extent. In summary, our model, trained on unpaired data, is capable of extracting and preserving crucial features and fine-grained details, automatically focusing on various image regions, and enhancing the understanding of the overall image structure. Through extensive experimentation on both datasets, our model outperforms other competing algorithms in terms of results.

Furthermore, in addition to visual presentations, this paper also evaluates the clinical significance through expert analysis. Figures 13 and 14 illustrate the 3D reconstructions, demonstrating that sCT preserves the original anatomical structures and maintains a certain continuity in internal organ tissues. Figures 15 and 16 showcase the distribution of our sCT under different doses compared to the real clinical dose. The results indicate that the dose difference between our sCT and the real CT is minimal, confirming the clinical applicability of our method.

While our approach outperforms other unsupervised learning models, the results on the Chest dataset remain relatively average. For future work, we plan to experiment with the state-of-the-art diffusion models in the image generation domain and further investigate 3D image generation capabilities.

## Conclusions

This study proposes an unsupervised learning model, IViT-CycleGAN, aiming to synthesize sCT from CBCT for future clinical practice. IViT-CycleGAN presents a U-net framework, which is built upon the ViT architecture within the generator. This framework leverages the U-net structure to effectively extract and retain crucial features and intricate details. Moreover, we enhance the ViT model by integrating a deep convolutional network and the self-attention mechanism of Transformer into the feed-forward neural network. The objective is to automatically prioritize information from various image locations during the image generation process, leading to a better comprehension of the overall image structure and emphasizing regions with finer details. Consequently, the generated images exhibit enhanced clarity and realism. To enhance the stability of model training, a gradient penalty is introduced to ensure minimal variations in network weights for minor changes in the model’s input. Additionally, an additional loss term is included in the generator loss to reinforce the consistency between the generated and source images by capturing their differences. The results demonstrate that IViT-CycleGAN outperforms other unsupervised learning models in terms of generating sCT, thus validating the clinical applicability and robustness of our model. In future clinical practice, this method can assist clinicians in developing radiotherapy treatment plans.

## Data Availability

The datasets used and/or analysed during the current study are available from the corresponding author on reasonable request.
